# (μ-Benzoato-κ^2^ *O*:*O*′)tris­[μ-*N*,*N*′-bis­(4-meth­oxy­phen­yl)formamidinato-κ^2^ *N*:*N*′]dimolybdenum(II) dichloro­methane monosolvate

**DOI:** 10.1107/S1600536811002558

**Published:** 2011-01-26

**Authors:** L.-J. Han

**Affiliations:** a Department of Chemistry, Tongji University, Shanghai 200092, People’s Republic of China

## Abstract

The title compound, [Mo_2_(C_15_H_15_N_2_O_2_)_3_(C_7_H_5_O_2_)]·CH_2_Cl_2_, has a quadruply bonded Mo_2_^4+^ unit equatorially coordinated by three *N*,*N*′-bis­(4-μ-meth­oxy­phen­yl)formamidinate (DAniF) ligands and one benzoate anion. The Mo—Mo bond length of 2.0881 (8) Å is typical for quadruply bonded species. The phenyl ring and the connected dimetal chelating ring (Mo_2_O_2_C) are nearly co-planar, making a dihedral angle of 3.24 (13)°. The dichloromethane solvent molecule is disordered over four sets of sites with occupancies of 0.3:0.3:0.2:0.2.

## Related literature

For Mo_2_(DAniF)_3_(OOCCH_3_), see: Cotton *et al.* (2003 ▶). For Mo_2_(DAniF)_4_, see: Lin *et al.* (1996 ▶). For Mo_2_(OOCC_6_H_5_)_4_, see: Cotton *et al.* (1978 ▶).
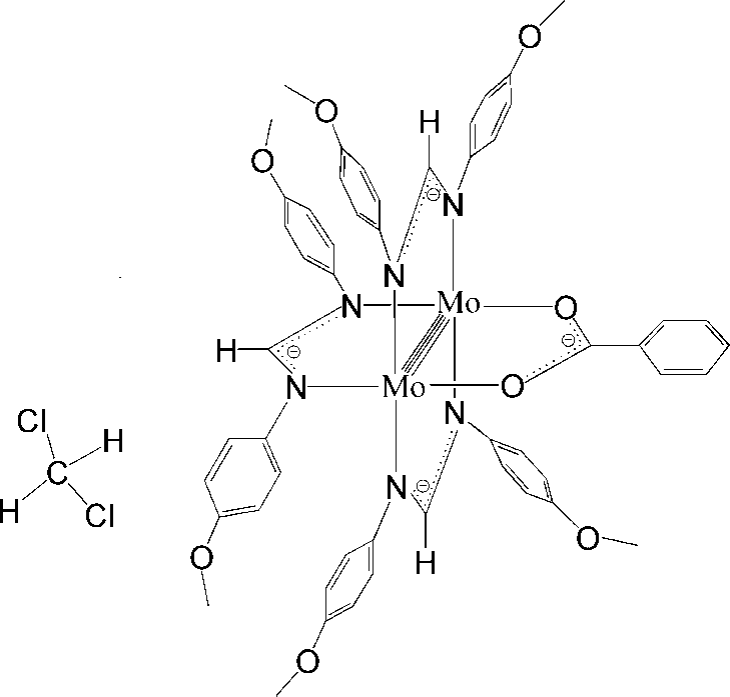


## Experimental

### 

#### Crystal data

[Mo_2_(C_15_H_15_N_2_O_2_)_3_(C_7_H_5_O_2_)]·CH_2_Cl_2_
*M* *_r_* = 1163.79
Triclinic, 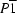

*a* = 12.025 (4) Å
*b* = 13.705 (4) Å
*c* = 17.727 (6) Å
α = 74.595 (4)°
β = 85.646 (4)°
γ = 67.238 (4)°
*V* = 2596.0 (14) Å^3^
*Z* = 2
Mo *K*α radiation
μ = 0.65 mm^−1^
*T* = 293 K
0.25 × 0.20 × 0.20 mm

#### Data collection

Bruker SMART 1000 diffractometer
Absorption correction: multi-scan (*SADABS*; Sheldrick, 2004 ▶) *T*_min_ = 0.851, *T*_max_ = 0.879
10844 measured reflections
8981 independent reflections
6852 reflections with *I* > 2σ(*I*)
*R*_int_ = 0.018

#### Refinement

*R*[*F* ^2^ > 2σ(*F* ^2^)] = 0.036
*wR*(*F* ^2^) = 0.107
*S* = 1.03
8981 reflections
652 parameters
12 restraints
H-atom parameters constrained
Δρ_max_ = 0.65 e Å^−3^
Δρ_min_ = −0.59 e Å^−3^

### 

Data collection: *APEX2* (Bruker, 2004 ▶); cell refinement: *SAINT-Plus* (Bruker, 2001 ▶); data reduction: *SAINT-Plus*; program(s) used to solve structure: *SHELXS97* (Sheldrick, 2008 ▶); program(s) used to refine structure: *SHELXL97* (Sheldrick, 2008 ▶); molecular graphics: *XP* in *SHELXTL* (Sheldrick, 2008 ▶); software used to prepare material for publication: *SHELXL97*.

## Supplementary Material

Crystal structure: contains datablocks global, I. DOI: 10.1107/S1600536811002558/om2393sup1.cif

Structure factors: contains datablocks I. DOI: 10.1107/S1600536811002558/om2393Isup2.hkl

Additional supplementary materials:  crystallographic information; 3D view; checkCIF report

## Figures and Tables

**Table 1:** Selected bond lengths (Å)

Mo1—N3	2.117 (3)
Mo1—N5	2.142 (3)
Mo1—O1	2.144 (2)
Mo1—N1	2.156 (3)
Mo2—N4	2.120 (3)
Mo2—N2	2.132 (3)
Mo2—O2	2.133 (2)
Mo2—N6	2.144 (3)

**Fig. 1.**
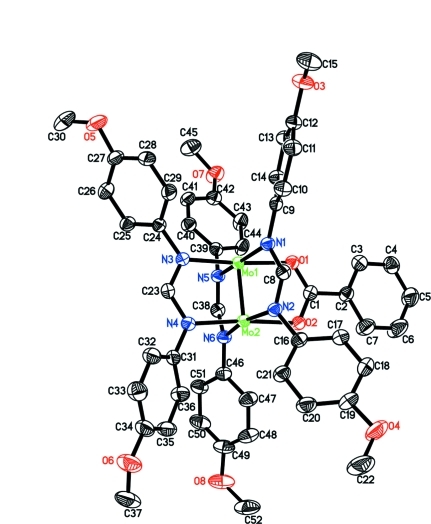
Molecular structure of the title compound drawn with displacement ellipsoids at the 30% probability level. The dichloromethane solvent molecule and hydrogen atoms have been omitted for clarity.

## supplementary crystallographic information

### Comment

Research on the quadruply bonded compounds has became an active areas in the field of crystal engineering, supramolecular chemistry and electronic communication. Different from single metal coordination building blocks, a covalent dimetal Mo_2_ unit has four equatorial positions as three-atom bridging ligand is used and two axial positions. In order to create designed molecules, nitrogen-donor ligand *N*,*N'*-di-*p*-anisylformamidinate (referred to as DAniF) is used to selectively block part of the equatorial coordination sites. Upon replacement of acetate in Mo_2_(DAniF)_3_(OOCCH_3_) by a benzoate group, compound Mo_2_(DAniF)_3_(OOCC_6_H_5_) has been synthesized. Crystals Mo_2_(DAniF)_3_(OOCC_6_H_5_)(CH_2_Cl_2_) were obtained by crystallized from CH_3_CH_2_OH/CH_2_Cl_2_ solution. The single-crystal structure is shown in Fig. 1. The title compound crystalizes in the P-1 space group with molecules lying on general positions in the unit cell. The asymmetric unit contains one complex molecule and additionally one solvent molecule of dichloromethane.

The Mo—Mo bond length in title compound of 2.0881 (8) Å is quite similar to those in the Mo_2_(DAniF)_4_ and Mo_2_(OOCC_6_H_5_)_4_ which are 2.0964 (5) and 2.0960 (1) Å, respectively, and is typical for quadruply bonded species. As designed, this molecule is close to that of its precursor Mo_2_(DAniF)_3_(OOCCH_3_) by having the same coordination sphere and a similar Mo—Mo bond length (2.0888 (9) Å). The average Mo—N distance (2.135 Å) is also similar to that of Mo_2_(DAniF)_3_(OOCCH_3_)(2.140 Å), indicating that the Mo—N covalent bond is unperturbed by the inductive effect of the remote substituents.

### Experimental

The title compound was synthesized by following reaction. To the yellow solution of Mo_2_(DAniF)_3_(OOCCH_3_) (0.512 g, 0.500 mmol) in 15 ml of THF, was added 1.0 ml NaOCH_3_ solution (0.5 *M* in methanol). After stirring for about 2 h, a colorless microcrystalline material was removed by filtration. To the filtrate was added an excess of benzoic acid (0.080 g, 0.650 mmol). Upon stirring, the color of the mixture immediately changed from yellow to orange yellow. After stirring at room temperature for an additional 1 h, the solvent was removed under vacuum, and the residue was washed with ethanol (2×15 ml), and then dried under vacuum. The orange yellow solid was dissolved in dichloromethane (15 ml) and the solution was layered with ethanol. Yellow block-shaped crystals formed after three days. Yield: 0.420 g (78%). ^1^HNMR(CDCl_3_, p.p.m.): 8.47(s,1*H*, –NCHN–), 8.37(s, 2H, –NCHN–), 8.29(t, 2H, aromatic), 7.46(t, 3H, aromatic), 6.63(d, 8H, aromatic), 6.58(d, 8H, aromatic), 6.44(d, 4H, aromatic), 6.23(d, 4H, aromatic), 3.71(s, 12H, –OCH_3_), 3.67(s, 12H, –OCH_3_). Anal. Calcd. C_52_H_50_Mo_2_N_6_O_8_: C, 57.89; H, 4.67; N, 7.79; Found: C, 57.07; H, 4.76; N, 7.59.

### Refinement

H atoms were positioned geometrically with C—H = 0.93, 0.97 and 0.96 Å, for aromatic, methylene, and methyl H atoms, respectively, and constrained to ride on their parent atoms, with *U*_iso_(H) = *xU*_eq_(C), where *x* = 1.5 for methyl H and *x* = 1.2 for aromatic H atoms. The dichloromethane solvent molecule exhibits high thermal motions and was refined as disordered over four sites with equivalent bonds from the disordered components restrained to have similar lengths. C—Cl and Cl—Cl distances were fixed to 1.78 and 2.85 Å, respectively. The same U_ij_ parameters were used for atoms C1s/C2s/C3s/C4s, Cl1/Cl3/Cl5/Cl7, Cl2/Cl4/Cl6/Cl8, leading to refined site occupancies of 0.09: 0.17: 0.22.

### Crystal data

**Table:**

[Mo_2_(C_15_H_15_N_2_O_2_)_3_(C_7_H_5_O_2_)]·CH_2_Cl_2_	*Z* = 2
*M**_r_* = 1163.79	*F*(000) = 1188
Triclinic, *P*1	*D*_x_ = 1.489 Mg m^−^^3^
Hall symbol: -P 1	Mo *K*α radiation, λ = 0.71073 Å
*a* = 12.025 (4) Å	Cell parameters from 6372 reflections
*b* = 13.705 (4) Å	θ = 2.4–26.3°
*c* = 17.727 (6) Å	µ = 0.65 mm^−^^1^
α = 74.595 (4)°	*T* = 293 K
β = 85.646 (4)°	Block, yellow
γ = 67.238 (4)°	0.25 × 0.20 × 0.20 mm
*V* = 2596.0 (14) Å^3^	

### Data collection

**Table:**

Bruker SMART 1000 diffractometer	8981 independent reflections
Radiation source: fine-focus sealed tube	6852 reflections with *I* > 2σ(*I*)
graphite	*R*_int_ = 0.018
ω scans	θ_max_ = 25.0°, θ_min_ = 1.7°
Absorption correction: multi-scan (*SADABS*; Sheldrick, 2004)	*h* = −14→14
*T*_min_ = 0.851, *T*_max_ = 0.879	*k* = −16→16
10844 measured reflections	*l* = −16→21

### Refinement

**Table:**

Refinement on *F*^2^	Primary atom site location: structure-invariant direct methods
Least-squares matrix: full	Secondary atom site location: difference Fourier map
*R*[*F*^2^ > 2σ(*F*^2^)] = 0.036	Hydrogen site location: inferred from neighbouring sites
*wR*(*F*^2^) = 0.107	H-atom parameters constrained
*S* = 1.03	*w* = 1/[σ^2^(*F*_o_^2^) + (0.048*P*)^2^ + 2.2558*P*] where *P* = (*F*_o_^2^ + 2*F*_c_^2^)/3
8981 reflections	(Δ/σ)_max_ = 0.001
652 parameters	Δρ_max_ = 0.65 e Å^−^^3^
12 restraints	Δρ_min_ = −0.59 e Å^−^^3^

### Special details

**Table:**

Geometry. All e.s.d.'s (except the e.s.d. in the dihedral angle between two l.s. planes) are estimated using the full covariance matrix. The cell e.s.d.'s are taken into account individually in the estimation of e.s.d.'s in distances, angles and torsion angles; correlations between e.s.d.'s in cell parameters are only used when they are defined by crystal symmetry. An approximate (isotropic) treatment of cell e.s.d.'s is used for estimating e.s.d.'s involving l.s. planes.
Refinement. Refinement of *F*^2^ against ALL reflections. The weighted *R*-factor *wR* and goodness of fit *S* are based on *F*^2^, conventional *R*-factors *R* are based on *F*, with *F* set to zero for negative *F*^2^. The threshold expression of *F*^2^ > σ(*F*^2^) is used only for calculating *R*-factors(gt) *etc*. and is not relevant to the choice of reflections for refinement. *R*-factors based on *F*^2^ are statistically about twice as large as those based on *F*, and *R*- factors based on ALL data will be even larger.

### Fractional atomic coordinates and isotropic or equivalent isotropic displacement parameters (Å^2^)

**Table:**

	*x*	*y*	*z*	*U*_iso_*/*U*_eq_	Occ. (<1)
Mo1	0.29773 (2)	0.27398 (2)	0.599717 (16)	0.03700 (8)	
Mo2	0.33531 (3)	0.20914 (2)	0.719867 (16)	0.03797 (9)	
O1	0.1144 (2)	0.37048 (19)	0.61915 (13)	0.0433 (6)	
O2	0.1550 (2)	0.29894 (19)	0.74747 (13)	0.0430 (6)	
O3	0.0341 (3)	0.1526 (2)	0.31737 (17)	0.0688 (8)	
O4	0.1310 (3)	−0.1329 (3)	1.00562 (19)	0.0998 (12)	
O5	0.6233 (3)	0.2150 (3)	0.26384 (17)	0.0873 (10)	
O6	0.8253 (3)	−0.2027 (3)	0.9482 (2)	0.0927 (11)	
O7	0.1843 (3)	0.7823 (2)	0.34264 (17)	0.0756 (9)	
O8	0.4791 (4)	0.3747 (3)	1.0234 (2)	0.1021 (12)	
N1	0.2318 (2)	0.1509 (2)	0.59293 (15)	0.0389 (6)	
N2	0.2713 (2)	0.0826 (2)	0.72517 (15)	0.0403 (7)	
N3	0.4753 (2)	0.1823 (2)	0.57165 (16)	0.0412 (7)	
N4	0.5168 (2)	0.1112 (2)	0.70350 (16)	0.0432 (7)	
N5	0.3333 (2)	0.4157 (2)	0.59902 (16)	0.0421 (7)	
N6	0.3733 (3)	0.3454 (2)	0.73067 (16)	0.0435 (7)	
C1	0.0832 (3)	0.3611 (3)	0.6901 (2)	0.0426 (8)	
C2	−0.04220 (18)	0.4275 (2)	0.70731 (14)	0.0478 (9)	
C3	−0.1242 (2)	0.4981 (2)	0.64675 (12)	0.0542 (10)	
H3A	−0.1017	0.5021	0.5950	0.065*	
C4	−0.2400 (2)	0.5626 (2)	0.66352 (16)	0.0662 (12)	
H4A	−0.2949	0.6098	0.6230	0.079*	
C5	−0.2737 (2)	0.5565 (3)	0.74086 (19)	0.0862 (16)	
H5A	−0.3512	0.5996	0.7521	0.103*	
C6	−0.1917 (3)	0.4859 (3)	0.80143 (13)	0.114 (2)	
H6A	−0.2142	0.4819	0.8532	0.137*	
C7	−0.0759 (2)	0.4214 (3)	0.78465 (13)	0.0836 (16)	
H7A	−0.0210	0.3742	0.8252	0.100*	
C8	0.2241 (3)	0.0832 (3)	0.6604 (2)	0.0415 (8)	
H8	0.1851	0.0360	0.6619	0.050*	
C9	0.1847 (3)	0.1445 (3)	0.52418 (19)	0.0384 (8)	
C10	0.1831 (3)	0.0494 (3)	0.5125 (2)	0.0488 (9)	
H10	0.2168	−0.0157	0.5510	0.059*	
C11	0.1329 (3)	0.0480 (3)	0.4454 (2)	0.0530 (9)	
H11	0.1303	−0.0167	0.4400	0.064*	
C12	0.0868 (3)	0.1430 (3)	0.3867 (2)	0.0506 (9)	
C13	0.0926 (3)	0.2379 (3)	0.3953 (2)	0.0511 (9)	
H13	0.0645	0.3016	0.3549	0.061*	
C14	0.1396 (3)	0.2391 (3)	0.4633 (2)	0.0470 (9)	
H14	0.1414	0.3042	0.4687	0.056*	
C15	0.0363 (5)	0.0556 (4)	0.3027 (3)	0.0888 (16)	
H15A	0.1183	0.0047	0.3043	0.133*	
H15B	0.0004	0.0723	0.2519	0.133*	
H15C	−0.0082	0.0238	0.3419	0.133*	
C16	0.2377 (3)	0.0263 (3)	0.7972 (2)	0.0436 (8)	
C17	0.1183 (4)	0.0429 (3)	0.8126 (2)	0.0559 (10)	
H17	0.0589	0.0922	0.7753	0.067*	
C18	0.0853 (4)	−0.0121 (4)	0.8822 (2)	0.0672 (12)	
H18	0.0046	−0.0013	0.8911	0.081*	
C19	0.1727 (4)	−0.0829 (4)	0.9381 (2)	0.0631 (11)	
C20	0.2916 (4)	−0.0983 (4)	0.9253 (2)	0.0645 (12)	
H20	0.3503	−0.1453	0.9637	0.077*	
C21	0.3242 (4)	−0.0435 (3)	0.8545 (2)	0.0530 (10)	
H21	0.4048	−0.0541	0.8459	0.064*	
C22	0.2169 (6)	−0.2152 (5)	1.0604 (3)	0.115 (2)	
H22A	0.2692	−0.2701	1.0357	0.172*	
H22B	0.1770	−0.2477	1.1025	0.172*	
H22C	0.2636	−0.1842	1.0807	0.172*	
C23	0.5519 (3)	0.1167 (3)	0.6304 (2)	0.0456 (9)	
H23	0.6304	0.0745	0.6203	0.055*	
C24	0.5180 (3)	0.1901 (3)	0.49368 (19)	0.0403 (8)	
C25	0.5999 (3)	0.2382 (3)	0.4668 (2)	0.0482 (9)	
H25	0.6301	0.2645	0.5004	0.058*	
C26	0.6380 (4)	0.2482 (3)	0.3903 (2)	0.0539 (10)	
H26	0.6937	0.2807	0.3730	0.065*	
C27	0.5935 (4)	0.2101 (3)	0.3401 (2)	0.0551 (10)	
C28	0.5139 (4)	0.1594 (4)	0.3678 (2)	0.0682 (12)	
H28	0.4858	0.1308	0.3348	0.082*	
C29	0.4754 (3)	0.1502 (3)	0.4434 (2)	0.0561 (10)	
H29	0.4204	0.1171	0.4607	0.067*	
C30	0.7083 (5)	0.2601 (4)	0.2326 (3)	0.0922 (17)	
H30A	0.6773	0.3360	0.2326	0.138*	
H30B	0.7242	0.2536	0.1799	0.138*	
H30C	0.7817	0.2217	0.2638	0.138*	
C31	0.5973 (3)	0.0305 (3)	0.7652 (2)	0.0459 (9)	
C32	0.6731 (4)	−0.0721 (3)	0.7584 (2)	0.0573 (10)	
H32	0.6738	−0.0905	0.7115	0.069*	
C33	0.7477 (4)	−0.1475 (4)	0.8199 (3)	0.0712 (13)	
H33	0.7977	−0.2165	0.8145	0.085*	
C34	0.7482 (4)	−0.1208 (4)	0.8895 (3)	0.0657 (12)	
C35	0.6727 (4)	−0.0204 (4)	0.8979 (2)	0.0653 (12)	
H35	0.6715	−0.0028	0.9452	0.078*	
C36	0.5979 (4)	0.0551 (3)	0.8359 (2)	0.0581 (10)	
H36	0.5471	0.1236	0.8419	0.070*	
C37	0.8501 (5)	−0.1717 (5)	1.0130 (3)	0.0993 (18)	
H37A	0.8781	−0.1129	0.9948	0.149*	
H37B	0.9111	−0.2331	1.0465	0.149*	
H37C	0.7779	−0.1479	1.0417	0.149*	
C38	0.3554 (3)	0.4280 (3)	0.6667 (2)	0.0446 (8)	
H38	0.3586	0.4935	0.6699	0.054*	
C39	0.2985 (3)	0.5108 (3)	0.5337 (2)	0.0413 (8)	
C40	0.3649 (3)	0.5121 (3)	0.4674 (2)	0.0509 (9)	
H40	0.4351	0.4517	0.4659	0.061*	
C41	0.3306 (3)	0.6007 (3)	0.4027 (2)	0.0535 (10)	
H41	0.3772	0.5995	0.3584	0.064*	
C42	0.2273 (4)	0.6906 (3)	0.4040 (2)	0.0522 (10)	
C43	0.1598 (4)	0.6910 (3)	0.4702 (3)	0.0635 (12)	
H43	0.0901	0.7517	0.4717	0.076*	
C44	0.1950 (4)	0.6019 (3)	0.5345 (2)	0.0599 (11)	
H44	0.1486	0.6031	0.5789	0.072*	
C45	0.2434 (5)	0.7782 (4)	0.2708 (3)	0.0832 (15)	
H45A	0.3262	0.7672	0.2779	0.125*	
H45B	0.2042	0.8458	0.2323	0.125*	
H45C	0.2399	0.7187	0.2535	0.125*	
C46	0.3980 (3)	0.3583 (3)	0.8035 (2)	0.0465 (9)	
C47	0.3522 (4)	0.3100 (4)	0.8706 (2)	0.0636 (11)	
H47	0.3031	0.2736	0.8657	0.076*	
C48	0.3765 (5)	0.3138 (4)	0.9441 (2)	0.0733 (13)	
H48	0.3452	0.2793	0.9879	0.088*	
C49	0.4469 (5)	0.3686 (4)	0.9525 (3)	0.0707 (13)	
C50	0.4929 (4)	0.4187 (4)	0.8872 (3)	0.0746 (13)	
H50	0.5396	0.4572	0.8925	0.090*	
C51	0.4704 (4)	0.4122 (3)	0.8137 (2)	0.0609 (11)	
H51	0.5043	0.4445	0.7703	0.073*	
C52	0.4277 (6)	0.3290 (5)	1.0907 (3)	0.107 (2)	
H52A	0.3417	0.3673	1.0875	0.161*	
H52B	0.4595	0.3354	1.1365	0.161*	
H52C	0.4469	0.2531	1.0941	0.161*	
Cl1	0.0625 (11)	0.6320 (9)	0.8223 (8)	0.212 (3)	0.30
Cl2	0.1659 (16)	0.6005 (13)	0.9619 (8)	0.201 (3)	0.30
C1S	0.087 (2)	0.7044 (19)	0.8855 (14)	0.231 (14)	0.30
H1S	0.0096	0.7329	0.9090	0.278*	0.30
H2S	0.1140	0.7638	0.8626	0.278*	0.30
Cl3	0.0397 (10)	0.5605 (9)	0.8530 (8)	0.212 (3)	0.30
Cl4	0.1674 (16)	0.6251 (13)	0.9421 (9)	0.201 (3)	0.30
C2S	0.1004 (11)	0.5307 (9)	0.9493 (7)	0.231 (14)	0.30
H3S	0.1621	0.4573	0.9629	0.278*	0.30
H4S	0.0368	0.5347	0.9869	0.278*	0.30
Cl5	0.1180 (11)	0.5692 (9)	0.7925 (7)	0.212 (3)	0.20
Cl6	0.0177 (11)	0.5259 (9)	0.9404 (7)	0.201 (3)	0.20
C3S	0.1202 (11)	0.5839 (9)	0.8918 (7)	0.231 (14)	0.20
H5S	0.0937	0.6603	0.8920	0.278*	0.20
H6S	0.2004	0.5444	0.9160	0.278*	0.20
Cl7	0.1116 (16)	0.5654 (14)	0.9890 (10)	0.212 (3)	0.20
Cl8	0.106 (2)	0.5768 (15)	0.8222 (10)	0.201 (3)	0.20
C4S	0.061 (4)	0.646 (3)	0.8957 (14)	0.231 (14)	0.20
H7S	−0.0221	0.6963	0.8899	0.278*	0.20
H8S	0.1102	0.6895	0.8870	0.278*	0.20

### Atomic displacement parameters (Å^2^)

**Table:**

	*U*^11^	*U*^22^	*U*^33^	*U*^12^	*U*^13^	*U*^23^
Mo1	0.03930 (16)	0.04008 (17)	0.03241 (15)	−0.01679 (13)	0.00142 (12)	−0.00815 (12)
Mo2	0.04070 (17)	0.04118 (17)	0.03255 (15)	−0.01694 (13)	0.00125 (12)	−0.00835 (12)
O1	0.0418 (13)	0.0437 (13)	0.0410 (13)	−0.0134 (11)	0.0021 (10)	−0.0101 (11)
O2	0.0425 (13)	0.0430 (13)	0.0390 (13)	−0.0123 (11)	0.0022 (10)	−0.0095 (10)
O3	0.0794 (19)	0.0722 (19)	0.0598 (17)	−0.0247 (16)	−0.0177 (15)	−0.0260 (15)
O4	0.105 (3)	0.118 (3)	0.0529 (19)	−0.042 (2)	0.0234 (18)	0.0085 (19)
O5	0.106 (2)	0.125 (3)	0.0418 (16)	−0.055 (2)	0.0204 (16)	−0.0279 (17)
O6	0.094 (2)	0.081 (2)	0.071 (2)	−0.010 (2)	−0.0320 (19)	0.0046 (18)
O7	0.086 (2)	0.0577 (18)	0.0575 (18)	−0.0157 (16)	0.0073 (16)	0.0073 (14)
O8	0.156 (3)	0.104 (3)	0.059 (2)	−0.054 (3)	−0.028 (2)	−0.0257 (19)
N1	0.0428 (15)	0.0406 (15)	0.0341 (14)	−0.0180 (13)	0.0009 (12)	−0.0075 (12)
N2	0.0452 (15)	0.0416 (16)	0.0350 (15)	−0.0200 (13)	0.0022 (12)	−0.0060 (12)
N3	0.0400 (15)	0.0493 (17)	0.0353 (15)	−0.0165 (13)	0.0029 (12)	−0.0136 (13)
N4	0.0401 (15)	0.0471 (17)	0.0374 (15)	−0.0126 (13)	−0.0022 (12)	−0.0083 (13)
N5	0.0468 (16)	0.0466 (16)	0.0366 (15)	−0.0239 (14)	0.0008 (12)	−0.0071 (13)
N6	0.0491 (16)	0.0468 (17)	0.0385 (15)	−0.0219 (14)	0.0001 (13)	−0.0115 (13)
C1	0.0450 (19)	0.0376 (18)	0.047 (2)	−0.0168 (16)	0.0060 (16)	−0.0132 (16)
C2	0.048 (2)	0.044 (2)	0.047 (2)	−0.0151 (17)	0.0056 (17)	−0.0103 (16)
C3	0.056 (2)	0.048 (2)	0.059 (2)	−0.0171 (19)	0.0017 (19)	−0.0184 (19)
C4	0.056 (2)	0.061 (3)	0.073 (3)	−0.017 (2)	−0.003 (2)	−0.011 (2)
C5	0.059 (3)	0.094 (4)	0.084 (4)	−0.007 (3)	0.013 (3)	−0.023 (3)
C6	0.076 (3)	0.141 (5)	0.065 (3)	0.013 (4)	0.020 (3)	−0.016 (3)
C7	0.069 (3)	0.092 (4)	0.055 (3)	0.001 (3)	0.005 (2)	−0.011 (3)
C8	0.0455 (19)	0.0405 (19)	0.0441 (19)	−0.0219 (16)	0.0029 (15)	−0.0119 (15)
C9	0.0341 (16)	0.0404 (18)	0.0409 (18)	−0.0140 (14)	0.0043 (14)	−0.0121 (15)
C10	0.056 (2)	0.042 (2)	0.047 (2)	−0.0174 (17)	−0.0045 (17)	−0.0087 (16)
C11	0.063 (2)	0.050 (2)	0.056 (2)	−0.0261 (19)	0.0000 (19)	−0.0227 (18)
C12	0.047 (2)	0.063 (2)	0.045 (2)	−0.0196 (18)	−0.0015 (16)	−0.0212 (18)
C13	0.058 (2)	0.046 (2)	0.046 (2)	−0.0159 (18)	−0.0047 (17)	−0.0095 (17)
C14	0.054 (2)	0.046 (2)	0.042 (2)	−0.0203 (17)	−0.0006 (16)	−0.0095 (16)
C15	0.110 (4)	0.096 (4)	0.084 (3)	−0.050 (3)	−0.017 (3)	−0.040 (3)
C16	0.055 (2)	0.0409 (19)	0.0387 (18)	−0.0223 (17)	0.0074 (16)	−0.0128 (15)
C17	0.056 (2)	0.067 (3)	0.044 (2)	−0.025 (2)	0.0016 (18)	−0.0084 (19)
C18	0.061 (3)	0.084 (3)	0.056 (3)	−0.032 (2)	0.016 (2)	−0.014 (2)
C19	0.080 (3)	0.065 (3)	0.043 (2)	−0.031 (2)	0.016 (2)	−0.0087 (19)
C20	0.073 (3)	0.063 (3)	0.043 (2)	−0.018 (2)	−0.002 (2)	−0.0013 (19)
C21	0.059 (2)	0.051 (2)	0.044 (2)	−0.0214 (19)	0.0063 (18)	−0.0057 (17)
C22	0.155 (6)	0.126 (5)	0.055 (3)	−0.073 (4)	0.003 (3)	0.020 (3)
C23	0.0365 (18)	0.053 (2)	0.046 (2)	−0.0158 (16)	0.0017 (16)	−0.0129 (17)
C24	0.0383 (17)	0.0429 (19)	0.0375 (18)	−0.0121 (15)	0.0029 (14)	−0.0126 (15)
C25	0.055 (2)	0.052 (2)	0.046 (2)	−0.0241 (18)	0.0062 (17)	−0.0213 (17)
C26	0.061 (2)	0.050 (2)	0.054 (2)	−0.0254 (19)	0.0149 (19)	−0.0165 (18)
C27	0.058 (2)	0.062 (2)	0.038 (2)	−0.016 (2)	0.0058 (17)	−0.0142 (18)
C28	0.067 (3)	0.108 (4)	0.051 (2)	−0.046 (3)	0.003 (2)	−0.037 (2)
C29	0.052 (2)	0.073 (3)	0.056 (2)	−0.033 (2)	0.0093 (18)	−0.026 (2)
C30	0.116 (4)	0.100 (4)	0.056 (3)	−0.042 (3)	0.034 (3)	−0.019 (3)
C31	0.0392 (18)	0.053 (2)	0.043 (2)	−0.0190 (17)	0.0004 (15)	−0.0066 (17)
C32	0.059 (2)	0.056 (2)	0.052 (2)	−0.015 (2)	−0.0056 (19)	−0.0140 (19)
C33	0.066 (3)	0.057 (3)	0.071 (3)	−0.004 (2)	−0.007 (2)	−0.014 (2)
C34	0.060 (3)	0.067 (3)	0.057 (3)	−0.017 (2)	−0.016 (2)	0.001 (2)
C35	0.073 (3)	0.073 (3)	0.044 (2)	−0.024 (2)	−0.010 (2)	−0.008 (2)
C36	0.068 (3)	0.053 (2)	0.046 (2)	−0.017 (2)	−0.0026 (19)	−0.0095 (18)
C37	0.106 (4)	0.105 (4)	0.068 (3)	−0.032 (3)	−0.042 (3)	0.009 (3)
C38	0.051 (2)	0.0424 (19)	0.047 (2)	−0.0237 (17)	0.0032 (16)	−0.0131 (16)
C39	0.0439 (18)	0.0410 (19)	0.0441 (19)	−0.0221 (16)	−0.0012 (15)	−0.0096 (15)
C40	0.053 (2)	0.049 (2)	0.047 (2)	−0.0148 (18)	0.0020 (17)	−0.0124 (17)
C41	0.059 (2)	0.060 (2)	0.041 (2)	−0.025 (2)	0.0095 (17)	−0.0110 (18)
C42	0.061 (2)	0.045 (2)	0.049 (2)	−0.0236 (19)	−0.0028 (18)	−0.0022 (17)
C43	0.056 (2)	0.049 (2)	0.067 (3)	−0.010 (2)	0.011 (2)	−0.003 (2)
C44	0.054 (2)	0.058 (2)	0.056 (2)	−0.019 (2)	0.0154 (19)	−0.0033 (19)
C45	0.114 (4)	0.064 (3)	0.055 (3)	−0.029 (3)	0.004 (3)	0.003 (2)
C46	0.058 (2)	0.0415 (19)	0.044 (2)	−0.0195 (17)	−0.0005 (17)	−0.0141 (16)
C47	0.086 (3)	0.070 (3)	0.050 (2)	−0.041 (2)	0.004 (2)	−0.022 (2)
C48	0.104 (4)	0.080 (3)	0.043 (2)	−0.039 (3)	0.008 (2)	−0.022 (2)
C49	0.097 (3)	0.066 (3)	0.051 (2)	−0.025 (3)	−0.019 (2)	−0.021 (2)
C50	0.097 (3)	0.071 (3)	0.069 (3)	−0.043 (3)	−0.025 (3)	−0.017 (2)
C51	0.074 (3)	0.067 (3)	0.051 (2)	−0.038 (2)	−0.009 (2)	−0.008 (2)
C52	0.139 (5)	0.111 (4)	0.052 (3)	−0.014 (4)	−0.013 (3)	−0.037 (3)
Cl1	0.174 (6)	0.163 (6)	0.279 (9)	−0.059 (5)	−0.030 (5)	−0.025 (6)
Cl2	0.225 (5)	0.196 (6)	0.188 (6)	−0.102 (4)	0.033 (4)	−0.034 (4)
C1S	0.096 (13)	0.27 (3)	0.38 (4)	−0.106 (17)	0.026 (17)	−0.11 (3)
Cl3	0.174 (6)	0.163 (6)	0.279 (9)	−0.059 (5)	−0.030 (5)	−0.025 (6)
Cl4	0.225 (5)	0.196 (6)	0.188 (6)	−0.102 (4)	0.033 (4)	−0.034 (4)
C2S	0.096 (13)	0.27 (3)	0.38 (4)	−0.106 (17)	0.026 (17)	−0.11 (3)
Cl5	0.174 (6)	0.163 (6)	0.279 (9)	−0.059 (5)	−0.030 (5)	−0.025 (6)
Cl6	0.225 (5)	0.196 (6)	0.188 (6)	−0.102 (4)	0.033 (4)	−0.034 (4)
C3S	0.096 (13)	0.27 (3)	0.38 (4)	−0.106 (17)	0.026 (17)	−0.11 (3)
Cl7	0.174 (6)	0.163 (6)	0.279 (9)	−0.059 (5)	−0.030 (5)	−0.025 (6)
Cl8	0.225 (5)	0.196 (6)	0.188 (6)	−0.102 (4)	0.033 (4)	−0.034 (4)
C4S	0.096 (13)	0.27 (3)	0.38 (4)	−0.106 (17)	0.026 (17)	−0.11 (3)

### Geometric parameters (Å, °)

**Table:**

Mo1—Mo2	2.0881 (8)	C28—C29	1.374 (5)
Mo1—N3	2.117 (3)	C28—H28	0.9300
Mo1—N5	2.142 (3)	C29—H29	0.9300
Mo1—O1	2.144 (2)	C30—H30A	0.9600
Mo1—N1	2.156 (3)	C30—H30B	0.9600
Mo2—N4	2.120 (3)	C30—H30C	0.9600
Mo2—N2	2.132 (3)	C31—C32	1.380 (5)
Mo2—O2	2.133 (2)	C31—C36	1.382 (5)
Mo2—N6	2.144 (3)	C32—C33	1.377 (5)
O1—C1	1.272 (4)	C32—H32	0.9300
O2—C1	1.269 (4)	C33—C34	1.378 (6)
O3—C12	1.378 (4)	C33—H33	0.9300
O3—C15	1.411 (5)	C34—C35	1.365 (6)
O4—C19	1.376 (5)	C35—C36	1.384 (5)
O4—C22	1.397 (6)	C35—H35	0.9300
O5—C27	1.362 (4)	C36—H36	0.9300
O5—C30	1.399 (6)	C37—H37A	0.9600
O6—C34	1.387 (5)	C37—H37B	0.9600
O6—C37	1.414 (6)	C37—H37C	0.9600
O7—C42	1.373 (4)	C38—H38	0.9300
O7—C45	1.414 (5)	C39—C40	1.370 (5)
O8—C49	1.375 (5)	C39—C44	1.382 (5)
O8—C52	1.412 (7)	C40—C41	1.379 (5)
N1—C8	1.326 (4)	C40—H40	0.9300
N1—C9	1.419 (4)	C41—C42	1.374 (5)
N2—C8	1.315 (4)	C41—H41	0.9300
N2—C16	1.424 (4)	C42—C43	1.376 (5)
N3—C23	1.322 (4)	C43—C44	1.379 (5)
N3—C24	1.429 (4)	C43—H43	0.9300
N4—C23	1.324 (4)	C44—H44	0.9300
N4—C31	1.431 (4)	C45—H45A	0.9600
N5—C38	1.314 (4)	C45—H45B	0.9600
N5—C39	1.437 (4)	C45—H45C	0.9600
N6—C38	1.334 (4)	C46—C47	1.386 (5)
N6—C46	1.416 (4)	C46—C51	1.388 (5)
C1—C2	1.487 (4)	C47—C48	1.376 (6)
C2—C3	1.3900	C47—H47	0.9300
C2—C7	1.3900	C48—C49	1.369 (7)
C3—C4	1.3900	C48—H48	0.9300
C3—H3A	0.9300	C49—C50	1.376 (6)
C4—C5	1.3900	C50—C51	1.383 (6)
C4—H4A	0.9300	C50—H50	0.9300
C5—C6	1.3900	C51—H51	0.9300
C5—H5A	0.9300	C52—H52A	0.9600
C6—C7	1.3900	C52—H52B	0.9600
C6—H6A	0.9300	C52—H52C	0.9600
C7—H7A	0.9300	Cl1—C1S	1.786 (18)
C8—H8	0.9300	Cl1—H5S	1.5004
C9—C10	1.380 (5)	Cl1—H7S	1.7118
C9—C14	1.394 (5)	Cl1—H8S	1.7870
C10—C11	1.382 (5)	Cl2—C1S	1.699 (18)
C10—H10	0.9300	Cl2—H5S	1.4265
C11—C12	1.377 (5)	Cl2—H6S	1.2080
C11—H11	0.9300	Cl2—H8S	1.5302
C12—C13	1.377 (5)	C1S—H1S	0.9700
C13—C14	1.376 (5)	C1S—H2S	0.9699
C13—H13	0.9300	C1S—H5S	0.5593
C14—H14	0.9300	C1S—H8S	0.2721
C15—H15A	0.9600	Cl3—C2S	1.790 (13)
C15—H15B	0.9600	Cl4—C2S	1.745 (14)
C15—H15C	0.9600	Cl4—H5S	1.1702
C16—C21	1.380 (5)	Cl4—H6S	1.2247
C16—C17	1.381 (5)	Cl4—H8S	1.1748
C17—C18	1.382 (5)	C2S—H3S	0.9699
C17—H17	0.9300	C2S—H4S	0.9701
C18—C19	1.374 (6)	Cl5—C3S	1.8275
C18—H18	0.9300	Cl6—C3S	1.7675
C19—C20	1.372 (6)	Cl6—Cl6^i^	2.11 (2)
C20—C21	1.394 (5)	Cl6—H3S	1.6434
C20—H20	0.9300	Cl6—H4S	0.9219
C21—H21	0.9300	C3S—H5S	0.9700
C22—H22A	0.9600	C3S—H6S	0.9701
C22—H22B	0.9600	Cl7—C4S	1.726 (19)
C22—H22C	0.9600	Cl7—H3S	1.5497
C23—H23	0.9300	Cl7—H4S	1.1378
C24—C25	1.377 (5)	Cl7—H6S	1.6270
C24—C29	1.379 (5)	Cl8—C4S	1.74 (2)
C25—C26	1.387 (5)	C4S—H1S	1.1946
C25—H25	0.9300	C4S—H5S	0.4944
C26—C27	1.374 (6)	C4S—H7S	0.9700
C26—H26	0.9300	C4S—H8S	0.9701
C27—C28	1.381 (6)		
			
Mo2—Mo1—N3	92.56 (7)	C36—C31—N4	118.7 (3)
Mo2—Mo1—N5	92.51 (7)	C33—C32—C31	121.0 (4)
N3—Mo1—N5	95.00 (11)	C33—C32—H32	119.5
Mo2—Mo1—O1	91.61 (6)	C31—C32—H32	119.5
N3—Mo1—O1	175.79 (9)	C32—C33—C34	120.0 (4)
N5—Mo1—O1	85.37 (10)	C32—C33—H33	120.0
Mo2—Mo1—N1	92.50 (7)	C34—C33—H33	120.0
N3—Mo1—N1	93.12 (11)	C35—C34—C33	119.9 (4)
N5—Mo1—N1	170.25 (10)	C35—C34—O6	124.4 (4)
O1—Mo1—N1	86.14 (10)	C33—C34—O6	115.7 (4)
Mo1—Mo2—N4	92.95 (8)	C34—C35—C36	119.8 (4)
Mo1—Mo2—N2	92.96 (7)	C34—C35—H35	120.1
N4—Mo2—N2	93.97 (11)	C36—C35—H35	120.1
Mo1—Mo2—O2	92.27 (6)	C31—C36—C35	121.2 (4)
N4—Mo2—O2	174.64 (10)	C31—C36—H36	119.4
N2—Mo2—O2	84.56 (10)	C35—C36—H36	119.4
Mo1—Mo2—N6	92.77 (8)	O6—C37—H37A	109.5
N4—Mo2—N6	95.24 (11)	O6—C37—H37B	109.5
N2—Mo2—N6	168.88 (11)	H37A—C37—H37B	109.5
O2—Mo2—N6	85.70 (10)	O6—C37—H37C	109.5
C1—O1—Mo1	116.5 (2)	H37A—C37—H37C	109.5
C1—O2—Mo2	116.6 (2)	H37B—C37—H37C	109.5
C12—O3—C15	117.3 (3)	N5—C38—N6	119.3 (3)
C19—O4—C22	117.3 (4)	N5—C38—H38	120.4
C27—O5—C30	118.7 (4)	N6—C38—H38	120.4
C34—O6—C37	117.2 (4)	C40—C39—C44	118.0 (3)
C42—O7—C45	117.1 (3)	C40—C39—N5	120.7 (3)
C49—O8—C52	117.2 (4)	C44—C39—N5	121.3 (3)
C8—N1—C9	118.2 (3)	C39—C40—C41	121.8 (4)
C8—N1—Mo1	116.2 (2)	C39—C40—H40	119.1
C9—N1—Mo1	125.3 (2)	C41—C40—H40	119.1
C8—N2—C16	117.3 (3)	C42—C41—C40	119.8 (3)
C8—N2—Mo2	117.2 (2)	C42—C41—H41	120.1
C16—N2—Mo2	121.4 (2)	C40—C41—H41	120.1
C23—N3—C24	118.4 (3)	O7—C42—C41	124.5 (4)
C23—N3—Mo1	117.5 (2)	O7—C42—C43	116.3 (4)
C24—N3—Mo1	124.0 (2)	C41—C42—C43	119.2 (3)
C23—N4—C31	118.5 (3)	C42—C43—C44	120.4 (4)
C23—N4—Mo2	117.0 (2)	C42—C43—H43	119.8
C31—N4—Mo2	124.1 (2)	C44—C43—H43	119.8
C38—N5—C39	117.5 (3)	C43—C44—C39	120.8 (4)
C38—N5—Mo1	117.7 (2)	C43—C44—H44	119.6
C39—N5—Mo1	121.9 (2)	C39—C44—H44	119.6
C38—N6—C46	119.3 (3)	O7—C45—H45A	109.5
C38—N6—Mo2	116.9 (2)	O7—C45—H45B	109.5
C46—N6—Mo2	123.1 (2)	H45A—C45—H45B	109.5
O2—C1—O1	123.0 (3)	O7—C45—H45C	109.5
O2—C1—C2	118.0 (3)	H45A—C45—H45C	109.5
O1—C1—C2	119.0 (3)	H45B—C45—H45C	109.5
C3—C2—C7	120.0	C47—C46—C51	116.5 (4)
C3—C2—C1	120.4 (2)	C47—C46—N6	118.7 (3)
C7—C2—C1	119.5 (2)	C51—C46—N6	124.7 (3)
C4—C3—C2	120.0	C48—C47—C46	122.6 (4)
C4—C3—H3A	120.0	C48—C47—H47	118.7
C2—C3—H3A	120.0	C46—C47—H47	118.7
C3—C4—C5	120.0	C49—C48—C47	119.7 (4)
C3—C4—H4A	120.0	C49—C48—H48	120.2
C5—C4—H4A	120.0	C47—C48—H48	120.2
C6—C5—C4	120.0	C48—C49—O8	124.2 (5)
C6—C5—H5A	120.0	C48—C49—C50	119.4 (4)
C4—C5—H5A	120.0	O8—C49—C50	116.4 (4)
C5—C6—C7	120.0	C49—C50—C51	120.5 (4)
C5—C6—H6A	120.0	C49—C50—H50	119.8
C7—C6—H6A	120.0	C51—C50—H50	119.8
C6—C7—C2	120.0	C50—C51—C46	121.2 (4)
C6—C7—H7A	120.0	C50—C51—H51	119.4
C2—C7—H7A	120.0	C46—C51—H51	119.4
N2—C8—N1	120.5 (3)	O8—C52—H52A	109.5
N2—C8—H8	119.8	O8—C52—H52B	109.5
N1—C8—H8	119.8	H52A—C52—H52B	109.5
C10—C9—C14	117.3 (3)	O8—C52—H52C	109.5
C10—C9—N1	124.2 (3)	H52A—C52—H52C	109.5
C14—C9—N1	118.6 (3)	H52B—C52—H52C	109.5
C9—C10—C11	122.0 (3)	C1S—Cl1—H7S	45.5
C9—C10—H10	119.0	H5S—Cl1—H7S	46.6
C11—C10—H10	119.0	H7S—Cl1—H8S	52.7
C12—C11—C10	119.6 (4)	C1S—Cl2—H6S	87.0
C12—C11—H11	120.2	H5S—Cl2—H6S	73.2
C10—C11—H11	120.2	H6S—Cl2—H8S	81.7
C11—C12—C13	119.5 (3)	Cl2—C1S—Cl1	102.0 (12)
C11—C12—O3	125.2 (4)	Cl2—C1S—H1S	100.5
C13—C12—O3	115.3 (3)	Cl1—C1S—H1S	104.0
C14—C13—C12	120.4 (3)	Cl2—C1S—H2S	120.3
C14—C13—H13	119.8	Cl1—C1S—H2S	118.3
C12—C13—H13	119.8	H1S—C1S—H2S	109.3
C13—C14—C9	121.1 (4)	Cl2—C1S—H5S	52.2
C13—C14—H14	119.4	Cl1—C1S—H5S	51.2
C9—C14—H14	119.4	H1S—C1S—H5S	99.8
O3—C15—H15A	109.5	H2S—C1S—H5S	151.0
O3—C15—H15B	109.5	Cl2—C1S—H7S	102.4
H15A—C15—H15B	109.5	Cl1—C1S—H7S	64.3
O3—C15—H15C	109.5	H2S—C1S—H7S	133.4
H15A—C15—H15C	109.5	H5S—C1S—H7S	70.8
H15B—C15—H15C	109.5	Cl2—C1S—H8S	47.9
C21—C16—C17	118.5 (3)	Cl1—C1S—H8S	85.8
C21—C16—N2	120.3 (3)	H1S—C1S—H8S	148.5
C17—C16—N2	121.2 (3)	H2S—C1S—H8S	91.1
C16—C17—C18	121.4 (4)	H5S—C1S—H8S	63.0
C16—C17—H17	119.3	H7S—C1S—H8S	133.7
C18—C17—H17	119.3	C2S—Cl4—H5S	71.8
C19—C18—C17	119.3 (4)	C2S—Cl4—H6S	50.7
C19—C18—H18	120.3	H5S—Cl4—H6S	82.5
C17—C18—H18	120.3	C2S—Cl4—H8S	96.3
C20—C19—C18	120.4 (4)	H6S—Cl4—H8S	97.8
C20—C19—O4	124.5 (4)	Cl4—C2S—Cl3	104.4 (7)
C18—C19—O4	115.1 (4)	Cl4—C2S—H3S	109.0
C19—C20—C21	119.8 (4)	Cl3—C2S—H3S	109.9
C19—C20—H20	120.1	Cl4—C2S—H4S	115.2
C21—C20—H20	120.1	Cl3—C2S—H4S	109.6
C16—C21—C20	120.5 (4)	H3S—C2S—H4S	108.6
C16—C21—H21	119.8	Cl3—C2S—H6S	88.3
C20—C21—H21	119.8	H3S—C2S—H6S	76.5
O4—C22—H22A	109.5	H4S—C2S—H6S	157.3
O4—C22—H22B	109.5	C3S—Cl6—Cl6^i^	133.0 (7)
H22A—C22—H22B	109.5	C3S—Cl6—H3S	62.1
O4—C22—H22C	109.5	Cl6^i^—Cl6—H3S	88.1
H22A—C22—H22C	109.5	C3S—Cl6—H4S	89.8
H22B—C22—H22C	109.5	H3S—Cl6—H4S	69.3
N3—C23—N4	120.0 (3)	Cl6—C3S—Cl5	103.1
N3—C23—H23	120.0	Cl6—C3S—H5S	111.1
N4—C23—H23	120.0	Cl5—C3S—H5S	111.2
C25—C24—C29	118.8 (3)	Cl6—C3S—H6S	111.1
C25—C24—N3	121.6 (3)	Cl5—C3S—H6S	111.2
C29—C24—N3	119.6 (3)	H5S—C3S—H6S	109.1
C24—C25—C26	121.1 (4)	Cl6—C3S—H8S	124.3
C24—C25—H25	119.5	Cl5—C3S—H8S	108.4
C26—C25—H25	119.5	H6S—C3S—H8S	98.8
C27—C26—C25	119.9 (4)	C4S—Cl7—H3S	93.5
C27—C26—H26	120.1	C4S—Cl7—H4S	85.7
C25—C26—H26	120.1	H3S—Cl7—H4S	69.8
O5—C27—C26	125.7 (4)	C4S—Cl7—H6S	60.7
O5—C27—C28	115.3 (4)	H3S—Cl7—H6S	55.1
C26—C27—C28	118.9 (4)	H4S—Cl7—H6S	110.0
C29—C28—C27	121.2 (4)	Cl7—C4S—Cl8	115.0 (17)
C29—C28—H28	119.4	Cl7—C4S—H1S	100.2
C27—C28—H28	119.4	Cl8—C4S—H1S	144.7
C28—C29—C24	120.1 (4)	Cl7—C4S—H5S	94.0
C28—C29—H29	119.9	Cl8—C4S—H5S	96.0
C24—C29—H29	119.9	H1S—C4S—H5S	78.6
O5—C30—H30A	109.5	Cl7—C4S—H7S	116.4
O5—C30—H30B	109.5	Cl8—C4S—H7S	113.6
H30A—C30—H30B	109.5	H1S—C4S—H7S	46.3
O5—C30—H30C	109.5	Cl7—C4S—H8S	100.1
H30A—C30—H30C	109.5	Cl8—C4S—H8S	102.8
H30B—C30—H30C	109.5	H1S—C4S—H8S	67.1
C32—C31—C36	118.0 (3)	H7S—C4S—H8S	106.6
C32—C31—N4	123.3 (3)		
			
N3—Mo1—Mo2—N4	0.09 (11)	O3—C12—C13—C14	177.8 (3)
N5—Mo1—Mo2—N4	−95.03 (11)	C12—C13—C14—C9	1.4 (6)
O1—Mo1—Mo2—N4	179.53 (10)	C10—C9—C14—C13	1.7 (5)
N1—Mo1—Mo2—N4	93.33 (11)	N1—C9—C14—C13	−179.8 (3)
N3—Mo1—Mo2—N2	−94.05 (11)	C8—N2—C16—C21	134.2 (4)
N5—Mo1—Mo2—N2	170.83 (10)	Mo2—N2—C16—C21	−69.0 (4)
O1—Mo1—Mo2—N2	85.40 (10)	C8—N2—C16—C17	−48.0 (5)
N1—Mo1—Mo2—N2	−0.81 (10)	Mo2—N2—C16—C17	108.8 (4)
N3—Mo1—Mo2—O2	−178.71 (10)	C21—C16—C17—C18	−2.7 (6)
N5—Mo1—Mo2—O2	86.17 (10)	N2—C16—C17—C18	179.5 (4)
O1—Mo1—Mo2—O2	0.73 (9)	C16—C17—C18—C19	1.6 (7)
N1—Mo1—Mo2—O2	−85.47 (10)	C17—C18—C19—C20	0.4 (7)
N3—Mo1—Mo2—N6	95.49 (11)	C17—C18—C19—O4	179.4 (4)
N5—Mo1—Mo2—N6	0.37 (10)	C22—O4—C19—C20	−8.5 (8)
O1—Mo1—Mo2—N6	−85.07 (10)	C22—O4—C19—C18	172.6 (5)
N1—Mo1—Mo2—N6	−171.27 (10)	C18—C19—C20—C21	−1.2 (7)
Mo2—Mo1—O1—C1	−0.9 (2)	O4—C19—C20—C21	179.9 (4)
N5—Mo1—O1—C1	−93.3 (2)	C17—C16—C21—C20	1.9 (6)
N1—Mo1—O1—C1	91.5 (2)	N2—C16—C21—C20	179.7 (4)
Mo1—Mo2—O2—C1	−0.9 (2)	C19—C20—C21—C16	0.1 (7)
N2—Mo2—O2—C1	−93.6 (2)	C24—N3—C23—N4	177.0 (3)
N6—Mo2—O2—C1	91.7 (2)	Mo1—N3—C23—N4	−0.8 (5)
Mo2—Mo1—N1—C8	5.5 (2)	C31—N4—C23—N3	172.9 (3)
N3—Mo1—N1—C8	98.2 (2)	Mo2—N4—C23—N3	0.9 (5)
O1—Mo1—N1—C8	−85.9 (2)	C23—N3—C24—C25	−68.3 (5)
Mo2—Mo1—N1—C9	178.4 (2)	Mo1—N3—C24—C25	109.3 (3)
N3—Mo1—N1—C9	−88.9 (2)	C23—N3—C24—C29	112.7 (4)
O1—Mo1—N1—C9	86.9 (2)	Mo1—N3—C24—C29	−69.7 (4)
Mo1—Mo2—N2—C8	−3.7 (2)	C29—C24—C25—C26	0.9 (5)
N4—Mo2—N2—C8	−96.9 (2)	N3—C24—C25—C26	−178.1 (3)
O2—Mo2—N2—C8	88.3 (2)	C24—C25—C26—C27	0.3 (6)
N6—Mo2—N2—C8	117.2 (5)	C30—O5—C27—C26	1.5 (7)
Mo1—Mo2—N2—C16	−160.4 (2)	C30—O5—C27—C28	−176.7 (4)
N4—Mo2—N2—C16	106.4 (3)	C25—C26—C27—O5	179.9 (4)
O2—Mo2—N2—C16	−68.4 (2)	C25—C26—C27—C28	−1.9 (6)
N6—Mo2—N2—C16	−39.5 (7)	O5—C27—C28—C29	−179.2 (4)
Mo2—Mo1—N3—C23	0.3 (3)	C26—C27—C28—C29	2.5 (7)
N5—Mo1—N3—C23	93.1 (3)	C27—C28—C29—C24	−1.3 (7)
N1—Mo1—N3—C23	−92.3 (3)	C25—C24—C29—C28	−0.4 (6)
Mo2—Mo1—N3—C24	−177.3 (3)	N3—C24—C29—C28	178.6 (4)
N5—Mo1—N3—C24	−84.6 (3)	C23—N4—C31—C32	−35.2 (5)
N1—Mo1—N3—C24	90.0 (3)	Mo2—N4—C31—C32	136.2 (3)
Mo1—Mo2—N4—C23	−0.5 (3)	C23—N4—C31—C36	146.2 (4)
N2—Mo2—N4—C23	92.7 (3)	Mo2—N4—C31—C36	−42.4 (4)
N6—Mo2—N4—C23	−93.6 (3)	C36—C31—C32—C33	−0.4 (6)
Mo1—Mo2—N4—C31	−172.0 (3)	N4—C31—C32—C33	−179.0 (4)
N2—Mo2—N4—C31	−78.9 (3)	C31—C32—C33—C34	−0.6 (7)
N6—Mo2—N4—C31	94.9 (3)	C32—C33—C34—C35	1.5 (7)
Mo2—Mo1—N5—C38	−5.5 (2)	C32—C33—C34—O6	179.2 (4)
N3—Mo1—N5—C38	−98.3 (3)	C37—O6—C34—C35	−16.9 (7)
O1—Mo1—N5—C38	85.9 (3)	C37—O6—C34—C33	165.5 (5)
Mo2—Mo1—N5—C39	−166.2 (2)	C33—C34—C35—C36	−1.5 (7)
N3—Mo1—N5—C39	101.1 (3)	O6—C34—C35—C36	−179.0 (4)
O1—Mo1—N5—C39	−74.7 (2)	C32—C31—C36—C35	0.5 (6)
Mo1—Mo2—N6—C38	4.6 (2)	N4—C31—C36—C35	179.1 (4)
N4—Mo2—N6—C38	97.8 (3)	C34—C35—C36—C31	0.5 (7)
N2—Mo2—N6—C38	−116.4 (5)	C39—N5—C38—N6	171.7 (3)
O2—Mo2—N6—C38	−87.5 (2)	Mo1—N5—C38—N6	10.2 (4)
Mo1—Mo2—N6—C46	174.9 (3)	C46—N6—C38—N5	179.5 (3)
N4—Mo2—N6—C46	−91.9 (3)	Mo2—N6—C38—N5	−9.8 (4)
N2—Mo2—N6—C46	53.9 (6)	C38—N5—C39—C40	121.3 (4)
O2—Mo2—N6—C46	82.8 (3)	Mo1—N5—C39—C40	−78.1 (4)
Mo2—O2—C1—O1	0.4 (4)	C38—N5—C39—C44	−61.1 (5)
Mo2—O2—C1—C2	−178.1 (2)	Mo1—N5—C39—C44	99.5 (4)
Mo1—O1—C1—O2	0.4 (4)	C44—C39—C40—C41	−0.4 (6)
Mo1—O1—C1—C2	178.9 (2)	N5—C39—C40—C41	177.3 (3)
O2—C1—C2—C3	179.1 (2)	C39—C40—C41—C42	0.1 (6)
O1—C1—C2—C3	0.6 (4)	C45—O7—C42—C41	7.4 (6)
O2—C1—C2—C7	1.3 (4)	C45—O7—C42—C43	−172.2 (4)
O1—C1—C2—C7	−177.3 (3)	C40—C41—C42—O7	−179.2 (4)
C7—C2—C3—C4	0.0	C40—C41—C42—C43	0.3 (6)
C1—C2—C3—C4	−177.8 (3)	O7—C42—C43—C44	179.2 (4)
C2—C3—C4—C5	0.0	C41—C42—C43—C44	−0.4 (7)
C3—C4—C5—C6	0.0	C42—C43—C44—C39	0.1 (7)
C4—C5—C6—C7	0.0	C40—C39—C44—C43	0.3 (6)
C5—C6—C7—C2	0.0	N5—C39—C44—C43	−177.4 (4)
C3—C2—C7—C6	0.0	C38—N6—C46—C47	144.1 (4)
C1—C2—C7—C6	177.8 (3)	Mo2—N6—C46—C47	−26.0 (5)
C16—N2—C8—N1	166.7 (3)	C38—N6—C46—C51	−39.0 (5)
Mo2—N2—C8—N1	9.0 (4)	Mo2—N6—C46—C51	150.9 (3)
C9—N1—C8—N2	176.9 (3)	C51—C46—C47—C48	−0.3 (6)
Mo1—N1—C8—N2	−9.7 (4)	N6—C46—C47—C48	176.8 (4)
C8—N1—C9—C10	−31.5 (5)	C46—C47—C48—C49	1.1 (7)
Mo1—N1—C9—C10	155.8 (3)	C47—C48—C49—O8	−178.1 (4)
C8—N1—C9—C14	150.2 (3)	C47—C48—C49—C50	−0.4 (7)
Mo1—N1—C9—C14	−22.5 (4)	C52—O8—C49—C48	−5.4 (7)
C14—C9—C10—C11	−3.6 (5)	C52—O8—C49—C50	176.7 (5)
N1—C9—C10—C11	178.0 (3)	C48—C49—C50—C51	−1.2 (7)
C9—C10—C11—C12	2.5 (6)	O8—C49—C50—C51	176.8 (4)
C10—C11—C12—C13	0.7 (6)	C49—C50—C51—C46	2.0 (7)
C10—C11—C12—O3	−179.7 (3)	C47—C46—C51—C50	−1.3 (6)
C15—O3—C12—C11	−5.4 (6)	N6—C46—C51—C50	−178.2 (4)
C15—O3—C12—C13	174.2 (4)	Cl6^i^—Cl6—C3S—Cl5	165.5 (8)
C11—C12—C13—C14	−2.6 (6)		

Symmetry codes: (i) −*x*, −*y*+1, −*z*+2.
